# Based on mutated aptamer-smartphone colorimetric detection of metronidazole in milk

**DOI:** 10.3389/fbioe.2024.1444846

**Published:** 2024-08-02

**Authors:** Sicheng Zhang, Yadi Qin, Jie Yuan, Yu Wang, Jun Yao, Minwei Zhang

**Affiliations:** ^1^ School of Pharmacy, Xinjiang Medical University, Urumqi, China; ^2^ School of Pharmacy, Xinjiang Second Medical College, Karamay, China; ^3^ Key Laboratory of Active Components and Drug Release Technology of Natural Medicines in Xinjiang, Xinjiang Medical University, Urumqi, China; ^4^ College Life Science and Technology, Xinjiang University, Urumqi, China

**Keywords:** aptamer, colorimetric, metronidazole, base mutation, smartphone

## Abstract

Excessive residue of metronidazole (MNZ) in food is harmful to the human body. There is an urgent demand to develop a portable tool for MNZ detection on-site. In this study, fifteen aptamers were prepared through targeted base mutation. Apt1-3 with the highest enrichment was chosen for further study. Its affinity was characterized by molecular docking simulation, AuNPs colorimetric assay, graphene oxide (GO) fluorescence assay, and exonuclease assay. Kd was determined by GO fluorescence assay (Kd: 92.60 ± 25.59 nM). Its specificity was also characterized by an exonuclease assay. A novel aptasensor was constructed by using the newly identified aptamer combined with the smartphone dark box. The principle of color change is caused by the aggregation state of AuNPs. Smartphones act as reading instruments. The detection can be completed in just a few seconds without the aid of instruments, achieving a detection limit of 0.15 nmol/mL and a range of 6.7–44.4 nmol/mL (*R* ^2^ = 0.9810). Therefore, the constructed smartphone colorimetric sensor based on mutant aptamers has important applications in food detection.

## 1 Introduction

Aptamers, which bind targets with high affinity and specificity, are short, single-stranded DNA or RNA sequences isolated from random oligonucleotide libraries through an *in vitro* approach called exponential enrichment (SELEX) (Ellington and Szostak, 1990; Tuerk and Gold, 1990). The unique advantages of low cost, easy modification, high stability, and long shelf life make them suitable for the construction of biosensors (Nimjee et al., 2005; Dunn et al., 2017). SELEX is a high-throughput method for screening aptamers. Unfortunately, the screened aptamers need to be modified to improve efficiency (Khoshbin and Housaindokht, 2021). Thus, there is room for improvement in sensitivity, specificity, and affinity in the aptamers acquired through the SELEX. In addition, aptamer optimization is extremely important. So far, the base mutation strategy is an effective method to enhance aptamer affinity. The base mutation changes the hydrogen bond and van der Waals forces between the molecule and the target substance, and it also affects the spatial structure of the aptamer (Ma et al., 2022; Manuel et al., 2022). However, the workload of a completely randomized mutation design would be cumbersome. It was shown that the aptamer stem-loop low-energy A-T bases transformed into high-energy G-C bases that could affect the affinity of the aptamer with the target, obtaining high-affinity aptamers (Kimoto et al., 2016). And single-base A-G and T-C mutations have the same effect (Sha et al., 2023).

Over the past three decades, the field of aptamer-based sensing has evolved considerably. Apt-based biosensor detection technologies are characterized by ease of operation, high sensitivity, and automation and have been widely used in the fields of food safety detection, cell physiology, drug targeting, and disease diagnosis (Kwon et al., 2014; Nguyen et al., 2018; Wang et al., 2019; Zhu et al., 2024). Aptamer biosensors include fluorescent aptasensors, colorimetric aptasensors, electrochemical sensors, and surface plasmon resonance aptasensors (Bhalla et al., 2016; Mehlhorn et al., 2018; Guo et al., 2021; Chen et al., 2024). Among these, the aptasensors constructed based on the colorimetric method have the advantage of visualization (Geleta, 2022). However, the naked eye is not sensitive enough to color changes, which limits the application of colorimetric sensors on POCT platforms (Kim and Cho, 2022). To solve this problem, a smartphone-based color-pickup device was designed. It is currently being used in paper-based colorimetry (Jiang et al., 2023), gold nanoparticle lateral flow assay (LFA) (Ruppert et al., 2019), disease diagnosis, environmental monitoring, food safety (Zahra et al., 2022), and other areas (Huang et al., 2021). This device is based on the principle of converting experimental data into RGB parameters. The RGB color model is an additive color model that superimposes red, green, and blue light in various ways to form various colors. In the RGB color model, any color in the tertiary color space can be specified by its color coordinates (Fernandes et al., 2020; Laddha et al., 2022). Thus, it was used to build colorimetric ranges (Ciaccheri et al., 2023). The RGB Color Picker software on your smartphone takes a digital image of the area to be measured, identifies subtle differences in the hue of the image, and performs chromaticity value analysis by determining a linear or non-linear relationship between the concentration of the sample and the chromaticity value. Therefore, this sensor built on a smartphone’s colorimetric methodology has significant value on Point of Care Testing (POCT) platforms (Zhang et al., 2020).

**FIGURE 1:**
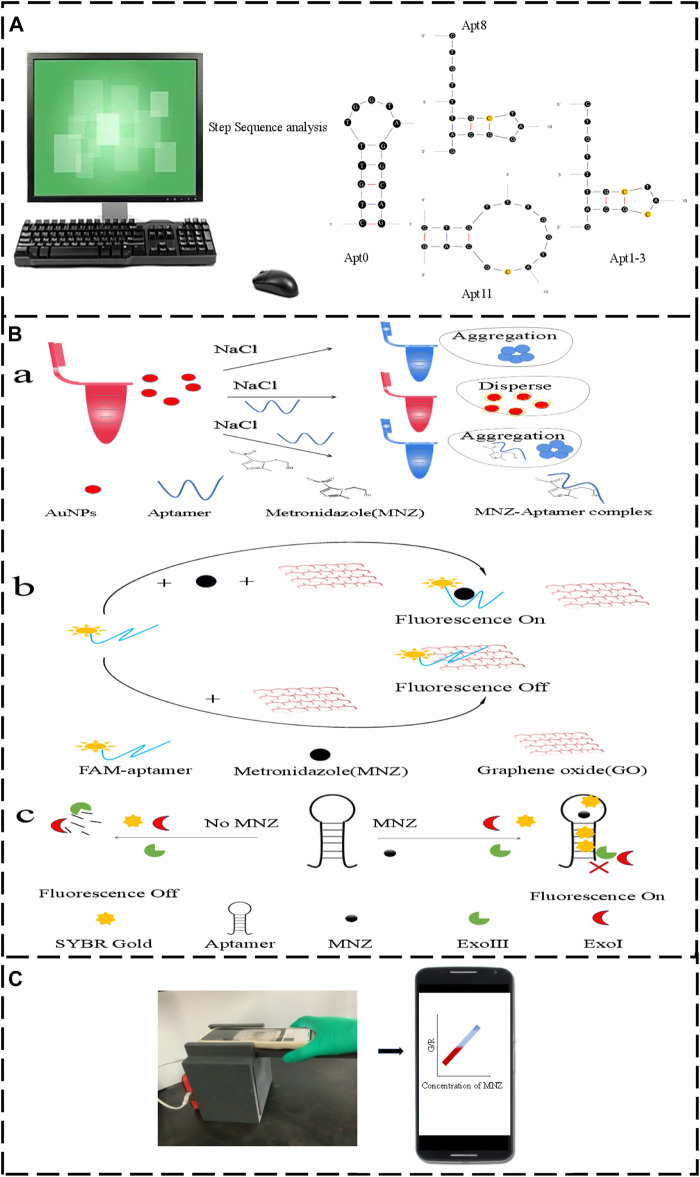
**(A)** The original aptamer-Apt0 was mutated to Apt8, Apt11 and Apt1-3. **(B,a)** Aptamer AuNPs schematic. **(B,b)** Graphene oxide fluorescence method schematic diagram. **(B,c)** Exonuclease schematic diagram. **(C)** Smartphone Dark box colorimetric device.

In this work, novel aptamers based on Apt0 were obtained by using single-base alterations in A-G, T-C, and C-G, G-C (Wei et al., 2020). Through secondary structure analysis and three-dimensional molecular docking, the binding and structural properties before and after the aptamer mutation were examined and characterized. Additionally, the binding affinity of MNZ to the aptamers was validated by exonuclease, GO fluorescence, and AuNPs colorimetry techniques (Figure 1). Then, the smartphone-based colorimetric aptasensor of MNZ was optimized using the aptamer with the greatest affinity. To raise the assay’s detection sensitivity under ideal detection circumstances. Using both original and mutant aptamers, the aptasensor’s sensitivity, specificity, and accuracy were evaluated. Lastly, the suggested colorimetric-sensitive smartphone sensor was used one more time to find the MNZ content in actual food samples. This work used the mutant aptamer to construct a fast and sensitive smartphone test based on the colorimetric approach to detect MNZ in milk. The assay has a lot of potential applications in the field of food safety detection.

## 2 Experimental

### 2.1 Reagents and materials

DNA probes (see Supplementary Tables S1, S2 for detailed sequences) were purchased from Shanghai Sangon Biotechnology Co., Ltd. (China). HAuCl4·3H2O, graphene oxide, ornidazole, tetracycline, norfloxacin, tobramycin sulfate, Tris-HCL (pH 6.8, pH 7.0, pH 7.4, pH 8.0, pH 8.8) buffer, acetonitrile, sodium carbonate, and NaCl were from Shanghai Macklin Biochemical Co., Ltd. (China). Metronidazole was purchased from Shanghai CATO Biochemical Co., Ltd. (China). Trisodium citrate dihydrate was obtained from Tianjin Yongsheng Fine Chemical Company (China). Dimethyl sulfoxide was purchased from Shanghai Biosharp Biochemical Co., Ltd. (China). Exonuclease Ⅰ and Exonuclease Ⅲ were from Wuhan ABclonal Biotechnology Co., Ltd. (China). SYBR Gold was obtained from Shanghai Thermo Fisher Scientific Co., Ltd. (China). Milk was obtained at the local market. All experimental water was Wahaha aquatic products in Hangzhou.

### 2.2 Apparatus

The UV-vis spectrophotometer SHIMADZU UV-2700 (SHIMADZU Co., Ltd.) was used. The transmission electron microscope JEM-2100 (JEOL Japan Electronics Co., Ltd.) was used. A chilled centrifuge, KDC-2044 (Zhongke Instrument Co., Ltd.), was used. An ultrasonic nicator, KQ3200DE (Kunshan Instrument Co., Ltd.), was used. Incubator shaker ZHWY2102C (Analytical Instrument Manufacturing Co., Ltd.) was used. Atomic Force Microscopy (AFM) Dimension ICON (Bruker AXS Co., Ltd.) was used. The fluorescence intensity was measured on an INFINITE 200Rro. (Tecan Trading Co., Ltd.). The mass weighing was obtained from the METTLER TOLEDO ABB5-S (Kaiwei Measurement Technology Co., Ltd.). The vortex mixer MS3BS25 (IKA Co., Ltd.) was used to fully mix the solution.

### 2.3 Characterization and theoretical validation of aptamer affinity

#### 2.3.1 MOE-docking simulation of aptamers bound to MNZ

The three-dimensional structure of the small molecule MNZ was obtained from the PubChem database (https://pubchem.ncbi.nlm.nih.gov). The chemicals that were downloaded were optimized and converted to mol2 format using Chem3D. Atomic charges and designated atom kinds were imported together with small molecule compounds into the Auto Dock Tools program. Every flexible key point has rotatable defaults. Ultimately, the optimal conformation was maintained as the docking ligand in the pdbqt format. Aptamer structures were predicted using the nucleic acid structure modeling service RNAComposer (https://rnacomposer.cs.put.poznan.pl/), which substitutes base T with U (Jeddi and Saiz, 2017). The molecular dynamics software Amber20 was used to repair the U of the aptamers’ structures to T. The force field used was Amber14SB, and it used minimal energy. In the end, it was kept as a docking aptamer. Using AutoDock4.2, the binding free energy of MNZ with aptamers was determined. The Lamarckian genetic technique was used to compute molecular docking. The final docking structure was assessed using binding free energy. Pymol 2.1 was used to process the docking findings.

#### 2.3.2 Molecular dynamics simulation

The Amber 2020 was used to perform molecular dynamics simulations of the aptamer-MNZ complexes. For the aptamers, the AMBER14SB force field characteristics were utilized, and for MNZ, the standard general force field parameters were employed. With atoms in the aptamer at least 1.0 nm away from the water box’s border, the TIP3P dominating water model was selected. Ions of either sodium or chloride were used to balance the system charge. The process of simulating molecular dynamics involved four stages: energy minimization, production kinetics simulation, equilibration, and heating. Using the MMPBSA approach, the free energy of binding between aptamer and MNZ was calculated.

### 2.4 Characterization and validation experiments of aptamer affinity

#### 2.4.1 Characterization of aptamer affinity based on colorimetric method validation

The following was the colorimetric analysis process: 300 μL of AuNPs and 200 μL of aptamer (0.06 μM) were combined and incubated at 25°C for 5 min. Following this, 200 µL of MNZ solution at several concentrations was added to the mixture, which was then reacted at 25°C for 25 min. Finally, 200 µL of 40 mM NaCl was added to mix evenly. The color of the solution was examined at various concentrations, and the spectrogram was quantified in the 400–800 nm ultraviolet spectrum region to determine a detectable linear range and LOD (LOD = 3σ/S, where σ is the standard deviation of the assay value for 11 blank samples and S is the linear slope).

#### 2.4.2 Characterization of aptamer affinity based on GO-based fluorescence method validation

The Kd values were determined with GO fluorescence (Chen et al., 2014). To measure the Kd values of aptamers binding to MNZ. The aptamer with FAM modified at the 5′end was denatured at 95 °C for 10 min, followed immediately by an ice bath for 10 min. Then, the 0–250 nM aptamer (100 μL) was shaken with 100 μL (1.5 μM) MNZ for 2 h. In the end, GO with a (GO/aptamer) mass ratio of 200/1 (200 μL, 2 mg/mL) was added, waiting 30 min to observe the fluorescence intensity. The fluorescence intensity was recorded (excitation and emission wavelengths were 485 nm and 520 nm). The affinity (Kd value) of the aptamer to MNZ can be effectively calculated. According to the equation ΔF = Bmax*ssDNA/(Kd + ssDNA) fitting by software Oring 2022 (ΔF = F - F0; F stands for the experimental group’s fluorescence intensity, F0 for the negative control group’s fluorescence intensity, ssDNA for the additional aptamer’s concentration, and Bmax for the maximum fluorescence intensity).

#### 2.4.3 Characterization of aptamer affinity based on exonuclease digestion method validation

Exonuclease digestion was used to further verify the affinity of aptamers (Alkhamis et al., 2023; Canoura et al., 2023). 100 μL aptamer and 100 μL MNZ (0.25 μM) were incubated at 25°C for 30 min, and 100 μL (15 U/mL) exonuclease was incubated at 25°C for 60 min. In the end, 300 µL mixed solution was injected into 100 μL (0.2 X SYBR Gold) and reacted at 25°C for 25 min. The fluorescence intensity was recorded (490 nm was the excitation wavelength and 540 nm was the emission wavelength).

### 2.5 Smartphone colorimetric analysis

A 3D dark box with an attached tape inside which was printed entirely in black polylactic acid (PLA) was designed to use CAD drawing combined with 3D printing technology. The box was lined with a black absorbent material. There, a smartphone dark box colorimetric device was established. For MNZ solution preparation, refer to Section 2.4.1. Colorimetric aptasensor. Firstly, the prepared MNZ solution was placed on the sample plate. Then, the sample plate was placed in the dark box. Finally, the smartphone was used for color. The G/R values were used to characterize the content of MNZ and to establish a detectable linear range with a limit of detection.

### 2.6 Selectivity assay

To test the proprietary nature of the MNZ sensing system, the system was tested using four MNZ analogs, tobramycin sulfate (TS), ornidazole (ONZ), norfloxacin (NFX) and tetracycline (Tet). These analogs were added to the established smartphone colorimetric sensor. Selective analysis based on the △R of G/R (△R = G/R with analogs - G/R with blank).

### 2.7 Application in milk

2 mL milk was prepared. Then 11.5 mL acetonitrile and trichloroacetic acid aqueous solution (9:1, v: v) were added to remove protein, and Na_2_CO_3_ aqueous solution (500 μL, 1.0 mol/L) was added to precipitate Ca^2 +^. Then shake for 10 min, ultrasound for 15 min, and stand for 20 min. Finally, The mixture was then centrifuged at 12,000 rpm for 15 min. The supernatant was transferred to an extraction column (HLB). The filtrate was collected and drynessed at 40°C under a stream of nitrogen. The residue was collected and dissolved for use.

## 3 Results and discussion

### 3.1 Characterization and theoretical validation of aptamer affinity

#### 3.1.1 Analysis of MOE-Docking results

Base mutations can alter the force of interaction between the aptamer and the target, thereby increasing the affinity of the aptamer (Ma et al., 2022; Manuel et al., 2022). Therefore, although the affinity and specificity of the original Apt0 were basically satisfactory, there is still potential to enhance its affinity by mutating specific bases.

**FIGURE 2:**
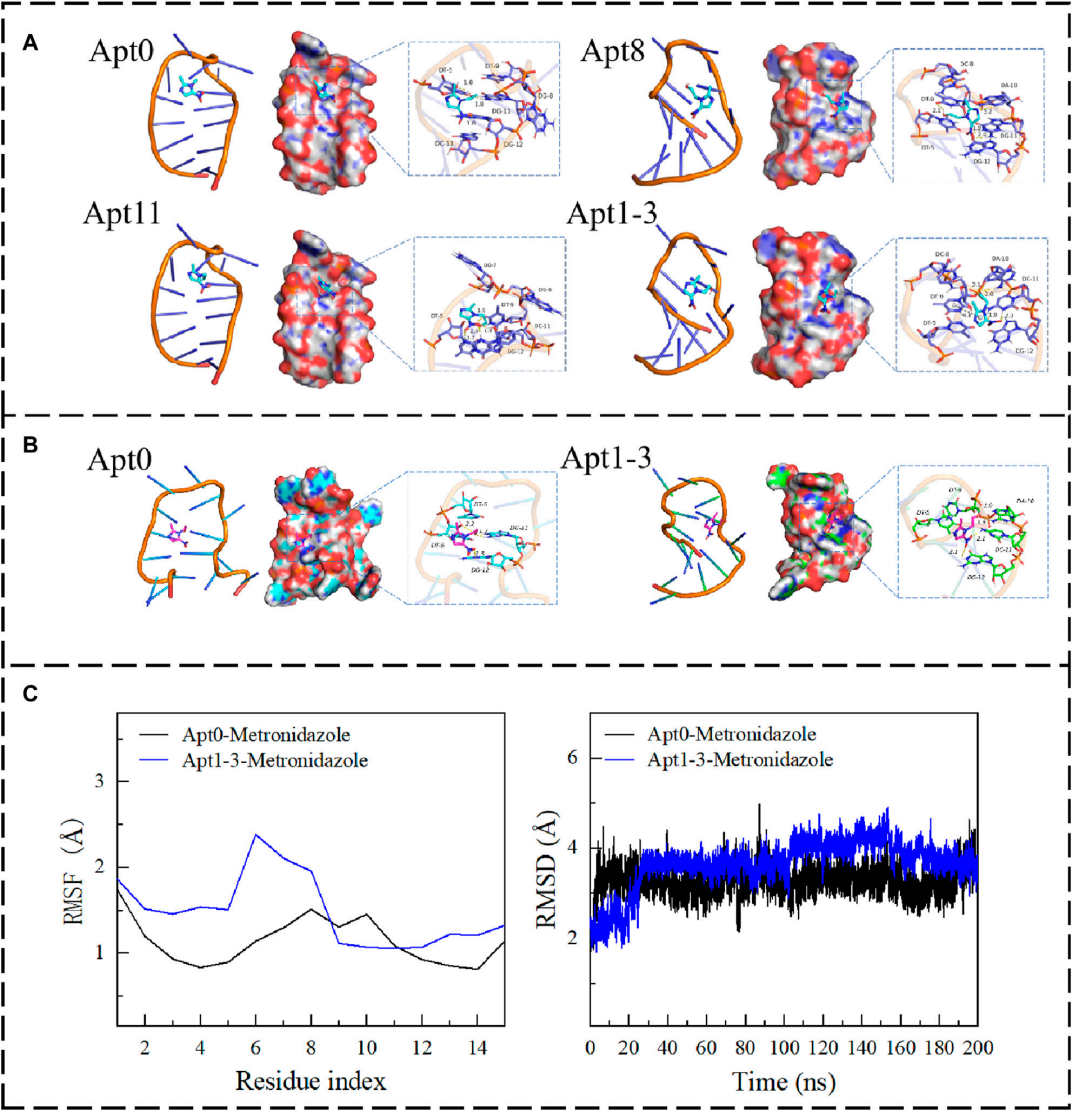
**(A)** The binding mode of Apt0, Apt8, Apt11, and Apt1-3 with MNZ. **(B)** The binding mode of Apt0 and Apt1-3 with MNZ after MD. **(C)** The RMSF and RMSD of Apt0 and Apt3 after 200 ns of molecular dynamics.

To reduce the cost of detection and improve affinity and performance, seven aptamers were obtained using A-G and T-C base mutations. To make a preliminary assessment of the affinity of the mutant aptamers, molecular docking was used to analyze the binding energies of the seven aptamers. The results showed that (Supplementary Table S3), the binding energy of the aptamers to MNZ was not greatly altered and was even lower than the original aptamer. Thus, eight new aptamers were obtained using C-G and G-C base mutations. The results showed that Apt8 and Apt11 had a good binding effect with MNZ, and the binding energies were −4.02 and −3.48 kcal/mol, respectively. Further, A new idea emerged: combining the superior Apt8 and Apt11 mutations to obtain a new aptamer, would its affinity be further enhanced? Therefore, a new Apt1-3 was obtained by combining Apt8 and Apt11. The results showed that Apt1-3 had a better binding effect with MNZ than Apt8 and Apt11, and the binding energy was −4.32 kcal/mol (Supplementary Table S3). The result was as expected. Next, the binding mechanisms of these four aptamers were further explored. The base residues that MNZ bound to the aptamer can be seen. The residues of MNZ interacting with Apt1-3 were C-8, A-10, T-9, T-5, G-12, C-11, etc. Among them, MNZ can interact with C-11 and A-10 through hydrogen bonds. There was a strong hydrogen bond interaction; the average hydrogen bond distance was 2.0 Å, and the length was less than 3.5 Å. It has a strong binding ability and plays an important role in stabilizing MNZ in the pocket of the Apt1-3 site. In addition, the five-membered nitrogen heterocyclic ring of MNZ formed a pi-pi conjugate interaction with the base part of the T-9 residue, which also made an important contribution to the stability of MNZ. Combined with Figure 2A, it can be found that MNZ matched well with the Apt1-3 site, which was conducive to the formation of a stable complex between MNZ and Apt1-3 (the secondary structures of the mutant aptamers are shown in Figure 1A; Supplementary Figure S1).

#### 3.1.2 Analysis of molecular dynamics results

To explore the changes in the affinity of the aptamers. The kinetic simulation was used to compare the original aptamer with the aptamer with the highest binding energy, which revealed all of them had changes in conformation for binding to MNZ, but all of them (Figure 2B) were able to form stable complexes with MNZ. The binding free energy was used for analyzing changes in aptamer binding modes by measuring the thermodynamic properties of the aptamer. Negative values of binding free energy (ΔGbinding energy) highlighted the stability of the system, while positive values showed instability. Electrostatic interactions had a high performance in stabilizing MNZ, followed by van der Waals interactions. The free energy of binding of MNZ to Apt1-3 aptamer was −28.52 ± −0.35 kJ/mol, where electrostatic occupied a major role (−22.32 ± −2.78 kcal/mol), which indicated that MNZ was able to remain stably in the aptamer site pocket with strong electrostatic interactions with the surrounding residues. In addition, MNZ formed effective van der Waals interactions with the surrounding residues (−17.25 ± −0.38 kcal/mol) due to a better fit to Apt1-3. The free energy of binding of MNZ to the Apt0 aptamer was −22.91 ± −0.76 kJ/mol, which was a weaker affinity than that of MNZ and Apt1-3, mainly because the electrostatic and van der Waals interactions between MNZ and Apt0 were weaker than those between MNZ and Apt1-3 (Figure 2C). In summary, MNZ and Apt1-3 had a strong affinity, and these affinities can lead to the formation of stable complexes between MNZ and aptamer, thus exerting active effects.

### 3.2 Characterization and validation experiments of aptamer affinity

#### 3.2.1 Colorimetric method validation

**FIGURE 3:**
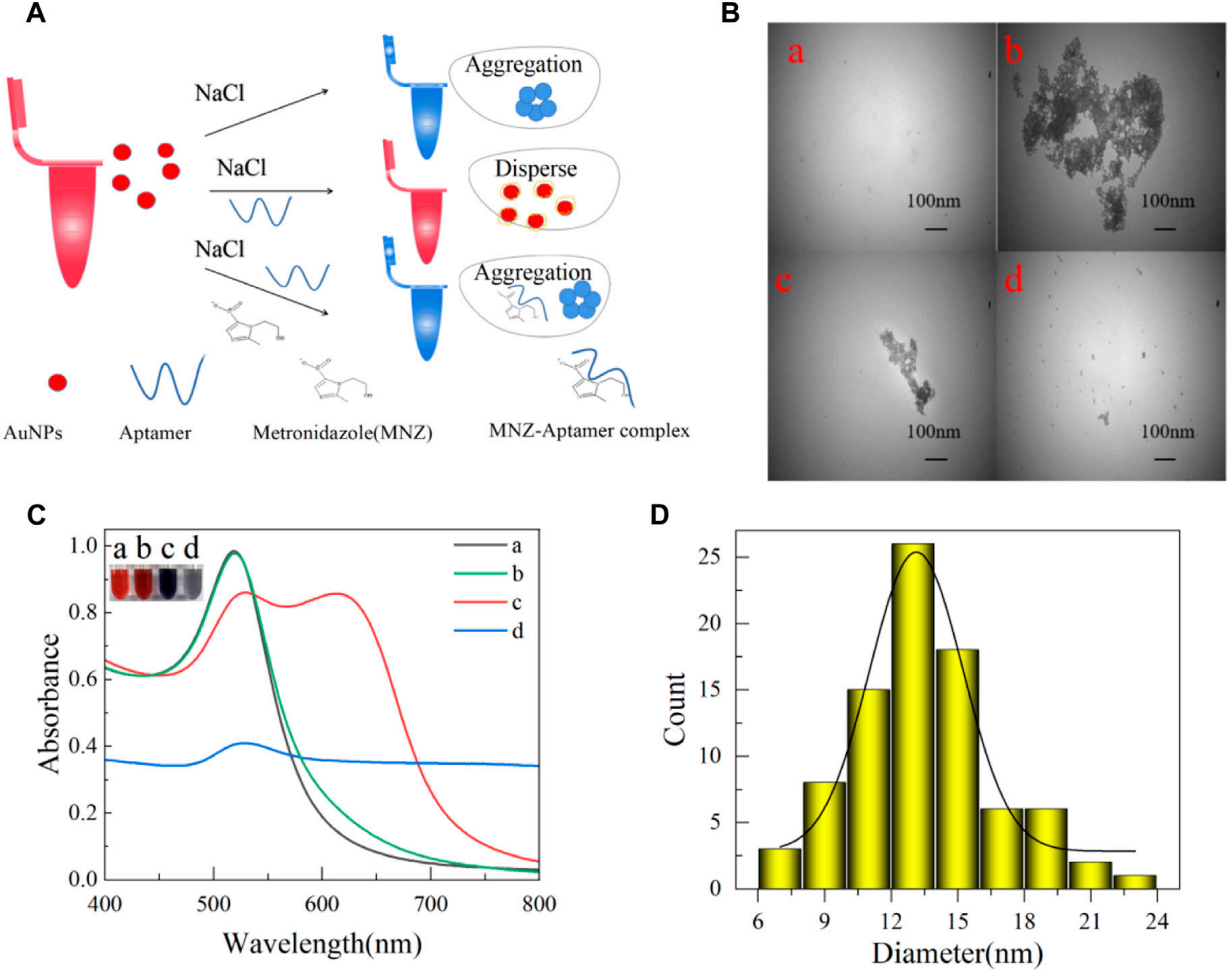
**(A)** AuNPs colorimetric schematic. **(B)** Transmission electron microscopy (TEM) of AuNPs in different states of 100 nm. **(C)** Spectrograms of AuNPs in different states (a: newly prepared AuNPs; b: 0.06 μM aptamer and 40 mM NaCl were added; c: added 0.06 μM aptamer, 40 μM MNZ, and 40 mM NaCl; d: added 40 mM NaCl). **(D)** AuNPs size distribution map.

The colorimetric method has been shown to measure aptamer affinity and is based on the fact that MNZ is bound specifically to the aptamer. It caused the AuNPs to aggregate at high NaCl concentrations. The solution would be from red to blue (Du et al., 2021) (Figure 3A). AuNPs were synthesized based on previous studies (Qin et al., 2022). In short, a 100 mL boiling solution of HAuCl4 (1 mM) was quickly injected with 10 mL of sodium citrate solution (38.8 mM) while being vigorously stirred. After 10 minutes of boiling and stirring, the heat was turned off, and the mixture was stirred for another 10 min. After reaching room temperature, the resultant wine-red solution was kept in dark glass bottles at 4°C for later use. AuNPs were subjected to transmission electron microscopy (TEM) scanning. From Figures 3B, C, its UV absorption peak is at 520 nm. According to Haiss et al. (Haiss et al., 2007; Zhao et al., 2008), it can be deduced that the molar concentration of AuNPs was approximately 3.52 × 10^−9^ mol/L. And, from Figure 3D, AuNPs had an average diameter of 13 nm. Overall, the above results indicate that the synthesis of AuNPs was successful.

The performance of the established MNZ test was significantly impacted by NaCl concentration, aptamer concentration, the incubation time of AuNPs and aptamer, the reaction time of aptamer and MNZ, the pH of Tris-HCL buffer solution, and temperature throughout incubation. However, AuNPs will aggregate with the increase in NaCl concentration, and the color will change from red to blue. Therefore, optimization of the concentration of NaCl was preferred for the reliability of the assay. Different concentrations of 200 μL of NaCl were added to 700 μL of AuNPs, and as the concentration of NaCl increased (Supplementary Figures S2A, B), the solution changed from red to blue, and the ratio of A650/A520 also increased. When the concentration of NaCl increased to 40 mM, the ratio was almost unchanged. The addition of NaCl at this point caused the AuNPs to aggregate almost completely, so the optimal concentration of NaCl was 40 mM. Then, to determine the optimal concentration of aptamer to protect AuNPs from high NaCl concentrations, 200 μL of aptamer of different concentrations was added to the AuNPs system. As the aptamer concentration increased, the solution changed from blue to red, and the ratio decreased. When the aptamer concentration increased to 0.06 μM, the ratio was almost unchanged (Supplementary Figures S2C, D). The amount of aptamer added at this point can completely adsorb and protect the surface of the AuNPs from high NaCl concentrations, so the optimal concentration was 0.06 μM. However, the protective effect was also influenced by the incubation time; with the increased incubation time of aptamer and AuNPs, the ratio decreased. When the incubation time increased to 5 min, the ratio tended to be stable, the aptamer was well absorbed on the surface of the AuNPs, and the incubation time was 5 min (Supplementary Figure S2E). Similarly, the reaction of MNZ with aptamer was also affected by time. As the reaction time of aptamer and MNZ increased, the ratio increased. When the time increased to 25 min, the ratio tended to be stable; MNZ had completely combined with the aptamer, so the incubation time was 25 min (Supplementary Figure S2F). The temperature and pH of the buffer will affect the system. For the temperature, with the increase in temperature in the system, the △R_A650/A520_ increased and then decreased. When the temperature was 25 °C, the △R_A650/A520_ was the highest. And the system was more sensitive. Therefore, 25 °C was selected as the optimal temperature (Supplementary Figure S2G). For the pH of the Tris-HCL buffer solution, with the increase of the pH of the Tris-HCL buffer solution of the aptamer in the system, the △R_A650/A520_ in the system increased first and then decreased. When pH was 7.0, the △R _A650/A520_ was the largest and most sensitive. Thus, the pH of the buffer solution was 7.0, which was the optimal pH (Supplementary Figure S2H) (△R_A650/A520_ = A_650/520_ with MNZ-A_650/520_ blank).

After the optimization of the experimental parameters to identify the high-affinity aptamer, the quantification performance of the MNZ colorimetric aptasensor was validated using a series of MNZ solutions with different concentrations, respectively. Among them, Apt8, Apt11, and Apt1-3 aptamers had a better performance. The UV-vis absorption spectrum of the MNZ colorimetric aptasensor against different MNZ concentrations was recorded in Supplementary Figure S3, S4 (other mutant aptamers of molecular docking binding energies and colorimetric results were analyzed in Supplementary Table S3). The aptasensor results showed that the detection line of Apt0 was 0.67 nmol/mL, Apt8 was 0.50 nmol/mL, Apt11 was 0.60 nmol/mL, and Apt1-3 was 0.12 nmol/mL. The aptasensors of Apt1-3 were nearly six times more sensitive compared to the aptasensors of Apt0. Consistent with the molecular docking results.

### 3.3 GO-based fluorescence measurement of dissociation constants

**FIGURE 4:**
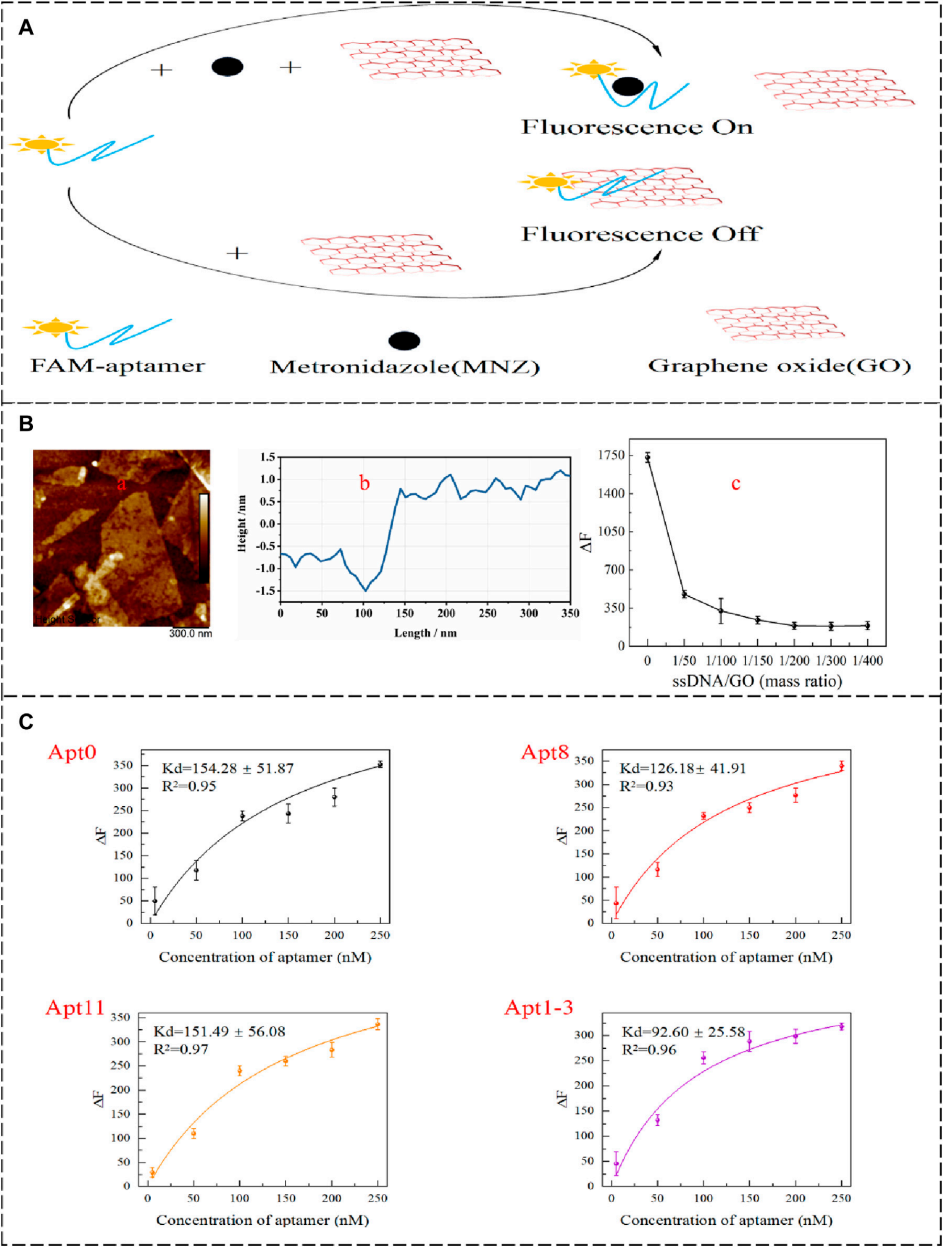
**(A)** GO-based fluorescence method to detect the affinity of aptamer schematic diagram. **(B,a,b)** The AFM images of GO. **(B,c)** Effect of GO addition concentration on △F. **(C)** Apt0, Apt8, Apt11, and Apt1-3 Kd value fitting curve.

To further validate the affinity, the GO fluorescence method was used to determine the dissociation constants (Xing et al., 2012). The 2D surface structure of GO and the excellent energy transfer (FRET) mechanism combine with aptamers to produce the effect of fluorescence quenching. The aptamer was not adsorbed by GO when the target was present (Figure 4A) (Zhang et al., 2017; Meng et al., 2022). To determine if the GO was a single layer. The characterization of the GO used from the AFM image (Figures 4B, a) showed that the thickness of the GO sheet was 2.55 nm (Figures 4B, b), which was within the expected range for monolayer GO. Meanwhile, the amount of GO added was optimized to make sure that the added GO could completely adsorb the aptamer that was not bound to the target. It can be seen from the figure (Figures 4B, c) that gradual fluorescence quenched with the addition of GO. When the mass ratio of the aptamer to GO is 1/50, the aptamer can be completely adsorbed by GO. Kd values of Apt0, Apt8, Apt11, and Apt1-3 were measured under optimized conditions. As the concentration of the aptamer increased, the fluorescence was enhanced, and the results are shown in Figure 4C. The content of the aptamer was directly proportional to the fluorescence intensity. Apt1-3 has a lower Kd value of 92.60 ± 25.58 nM, which means it has the highest affinity for MNZ.

### 3.4 Quantifying aptamer binding affinity using exonucleases

**FIGURE 5:**
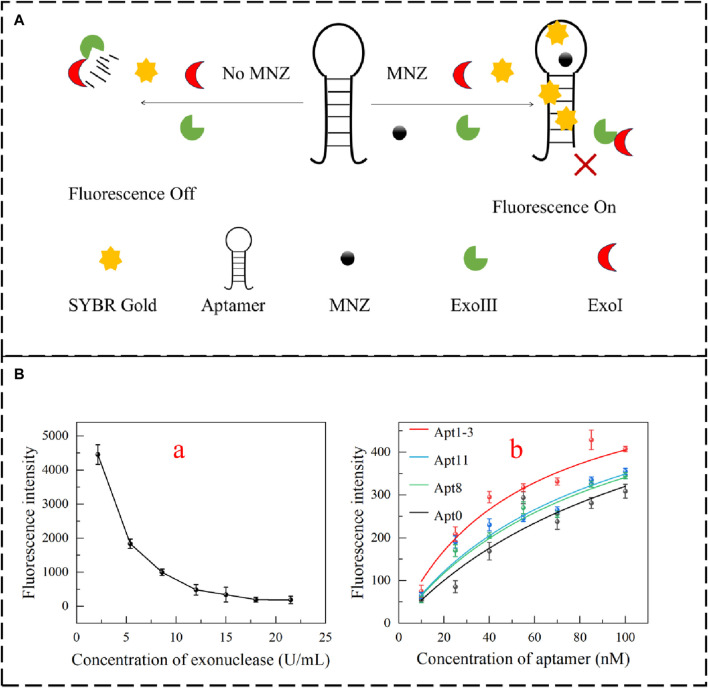
**(A)** Schematic diagram for detecting the affinity of aptamers based on the exonuclease fluorescent method. **(B,a)** Optimization of exonuclease incorporation. **(B,b)** Apt0, Apt8, Apt11 and Apt1-3 fluorescence intensity fitting.

To verify the affinity of the aptamer even further. Exonuclease digestion can be used to assess aptamer affinity (Alkhamis et al., 2023; Canoura et al., 2023). Exonucleases can digest aptamers, and the digested single-stranded DNA cannot be stained. In the presence of the target, it can prevent the digestion of aptamers. SYBR Gold can dye single-stranded DNA and show strong fluorescence. The affinity of the aptamer can be evaluated by this fluorescence alteration (Figure 5A). In this experiment, exonuclease Ⅰ and exonuclease Ⅲ were in equal proportion optimized (Figures 5B, a). Different concentrations of exonuclease (100 μL) were added to the 100 nM aptamer. The fluorescence intensity decreased significantly. When the concentration of exonuclease was increased to 15 U/mL, the fluorescence intensity hardly changed. It demonstrated that the aptamer was completely digested. When MNZ (0.25 μM) was added, as the concentration of aptamer increased, the fluorescence intensity was enhanced, and the results showed that Apt1-3 has a higher affinity for MNZ (Figures 5B, b).

### 3.5 Smartphone colorimetric

**FIGURE 6:**
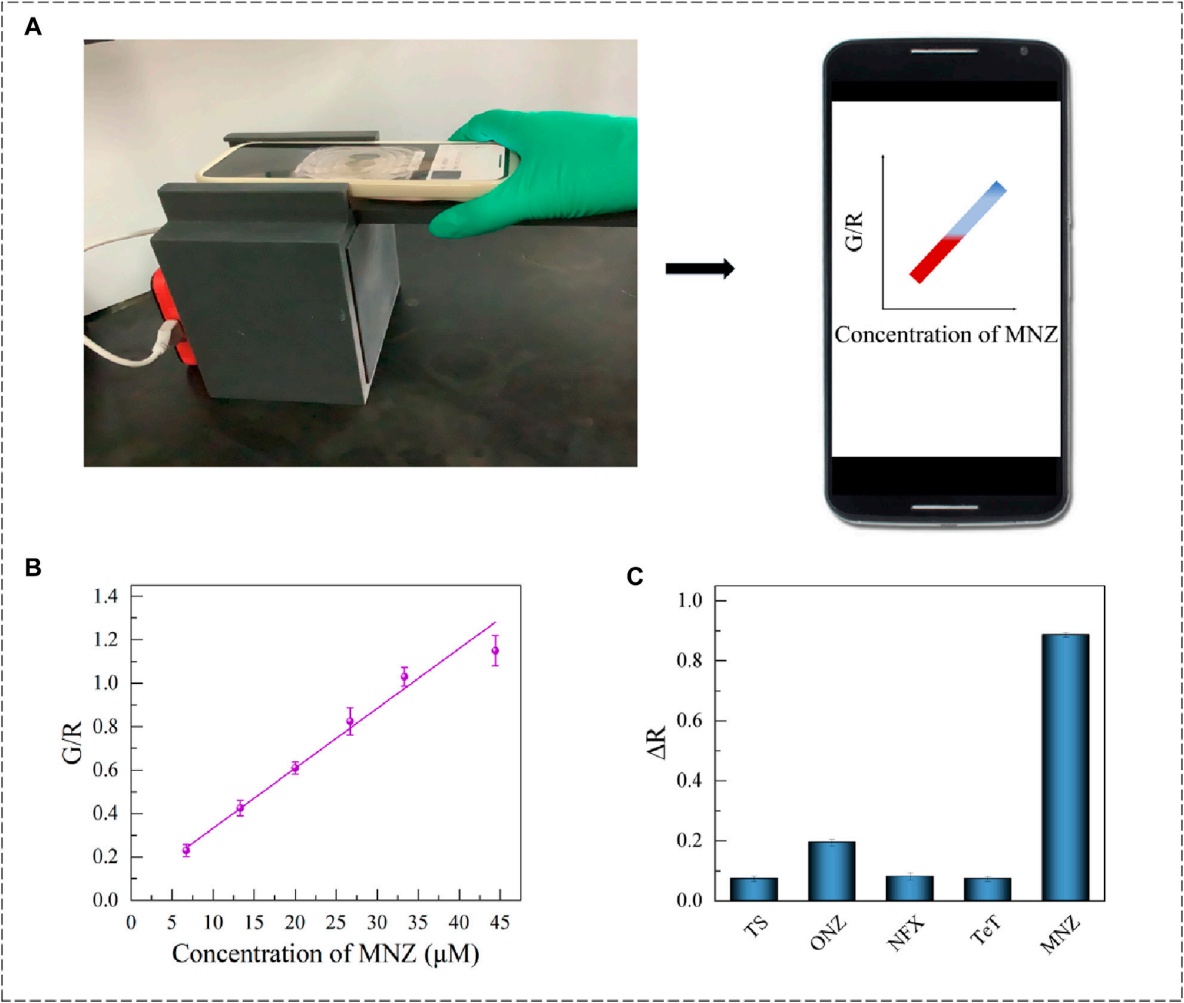
**(A)** Smartphone colorimetric device diagram and chart. **(B)** Apt1-3 Smartphone colorimetric linear range. **(C)** Apt1-3 proprietary validation chart.

To improve the convenience of the testing method, an RGB model-based smartphone device was established. It read the color change of the detection system and worked by analyzing the RGB value through smartphone software. Besides, to avoid the effects of environmental factors, 3D printing technology was used to design a dark box with tape inside, which was completely printed with black polylactic acid. The dark box was lined with black absorbing material, which can block the internal random reflection from visible light to infrared light (250–2000 nm), and the hemisphere reflectivity was less than 1%. It can effectively prevent the interference of stray light and internal reflection in the color selection process. The design drawing of the 3D dark box and its physical display are shown in Figure 6A and Supplementary Figure S5. It was perfectly combined with the smartphone colorimetric software F Color Picker (version 1.2.2). Different concentrations of MNZ were added to the sample plate for color-taking and counting. The experimental results showed that the G/R value was linearly related to the concentration of MNZ in the range 6.7–44.4 nmol/mL (*R* ^2^ = 0.9810), and the limit of detection (LOD) was 0.15 nmol/mL (Figure 6B). The final concentration obtained from the proposed smartphone platform was in good agreement with the UV result.

#### 3.5.1 Selective and real sample testing of smartphone

To investigate the specificity of the established installation, 200 μL (20 μM) of tobramycin sulfate (TS), ornidazole (ONZ), norfloxacin (NFX), and tetracycline (Tet) were added to the smartphone sensor system separately. After spass software calculation and analysis (*p* < 0.05) (Figure 6C), it was suggested that the proposed smartphone colorimetry assay presented excellent selectivity to distinguish MNZ from its analogues.

To confirm the feasibility of the smartphone sensor in real samples, 10 μM, 20 μM, and 30 μM MNZ were spiked into the milk sample. The spiked recoveries can be calculated according to the equation: spiked recovery = (spiked sample measured value - sample measured value)/spiked volume*100%. The results are presented in Table 1. The recoveries of Apt1-3 were in the range of 92.5%–100.4%, which demonstrated that the developed assay showed satisfactory performance for the detection of MNZ in real samples. Compared with the existing detection methods (see Table 2), the designed detector performed well. It is noteworthy that the actual concentration output can be accomplished in seconds without tedious steps. The method offers potential applications for POCT platforms.

**TABLE 1:** Recovery of Apt1-3 in milk.

Sample	Added(nmol/mL)	Recovery(%)
milk(Apt1-3)	10	(96.4 ± 3.1)
	20	(92.5 ± 2.3)
	30	(100.4 ± 2.0)

**TABLE 2:** Comparison table of metronidazole assay methods.

Methods	Linear range	LOD	Applications	(Mean)Recovery	Ref
Electrochemical sensors	0.4–500 μM	0.25 μM	Honey, egg	97.79%–104.42% 96.77%–103.34%	Li et al. (2023)
Carbon dots-based sensor	0–10 μg/mL	0.257 μg/mL	Honey, metronidazole tablets	98.0%–105.1% 95.7%–106.5%	Zhao et al. (2018)
Surface enhanced Raman spectroscopy	0–50 μg/mL	10.0 μg/mL	environmental Samples	-	Han et al. (2014)
High performance liquid chromatography (HPLC)	1.0–100.0 mg/L	1.0 mg/L	Calf Serum	93%	Holt et al. (1990)
Gas chromatograph	0.2–2.0 μg/kg	0.2 μkg/kg, 0.1 μkg/kg	Chicken porcine liver	72%, 89%	Ho et al. (2005)
LC-mass spectrometry (LC-MS/MS)	10–136.2 ng/L, 1.0–12.0 ng/g, 1.0–1.5 ng/g	3.4 ng/L, 0.4 ng/g, 0.3 ng/g	Water Sediment samples Tissue samples	88.0%–106.0% 95.2%–113.0% 98.0%–103.6%	Wagil et al. (2015)
Smartphone colourimetry	6.7–44.4 nmol/mL	0.15 nmol/mL	Milk	92.5%–100.4%	This assay

## 4 Conclusion

In this study, 15 aptamers were obtained by base-directed mutation using computer simulation, the colorimetric method, the GO fluorescence method, and the nucleic acid exonuclease method to find the Apt1-3 aptamer with high affinity. In addition, we present a portable, low-cost biosensor based on the combination of a smartphone and a 3D box for the detection of MNZ in milk. The determination of MNZ was achieved by analyzing the RGB values through the software F color picker (version 1.2.2) on the smartphone. This portable device has no restriction on the place of use. All the experimental results showed that this portable biosensor has a good ability to detect MNZ with an LOD of 0.15 nmol/mL. It demonstrates the great potential for drug residue detection. In conclusion, smartphone colorimetry will be applied in the biomedical field with its unique advantages and bring great benefits to mankind.

## Data Availability

The original contributions presented in the study are included in the article/Supplementary Material, further inquiries can be directed to the corresponding authors.
