# Supplementary material for: Based on mutated aptamer-smartphone colorimetric detection of metronidazole in milk
Source: Front Bioeng Biotechnol. 2024 Aug 2;12:1444846. doi: 10.3389/fbioe.2024.1444846 (PMC11327025; doi:10.3389/fbioe.2024.1444846)
Supplement: Supplementary file 1 [file DataSheet1.docx]

Supporting Information for

**Based on mutated aptamer-smartphone colorimetric detection of metronidazole in milk**

Sicheng Zhang^1^, Yadi Qin^1^, Jie Yuan^1,2^, Yu Wang^1^, Jun Yao^1,3^^[[1]](#footnote-0)^﹡and Minwei Zhang^4^^[[2]](#footnote-1)^﹡

*^[1]School of Pharmacy, Xinjiang Medical University, 830017 Shangde North Road Urumqi, China.^*

*^[2]School of Pharmacy, Xinjiang Second Medical College, 834000 Shengli Road Karamay, China.^*

*^[3]Key Laboratory of Active Components and Drug Release Technology of Natural Medicines in Xinjiang, Xinjiang Medical University, 830017 Shangde North Road Urumqi, China.^*

*^[4]College life science & technology, Xinjiang University, 830046 Shengli Road Urumqi, China.^*

**Table S1** The original and mutant aptamers, mutant bases labeled X.

| Aptamer number | Sequence(5'-3') |
| --- | --- |
| Apt0  Apt1  Apt2  Apt3  Apt4  Apt5  Apt6  Apt7  Apt8  Apt9  Apt10  Apt11  Apt12  Apt13  Apt14  Apt15  Apt1-3 | CTG TTT GGT AGG CAG  GTG TTT GGT AGG CAG  CCG TTT GGT AGG CAG  CTC TTT GGT AGG CAG  CTG CTT GGT AGG CAG  CTG TCT GGT AGG CAG  CTG TTC GGT AGG CAG  CTG TTT CGT AGG CAG  CTG TTT GCT AGG CAG  CTG TTT GGC AGG CAG  CTG TTT GGT GGG CAG  CTG TTT GGT ACG CAG  CTG TTT GGT AGC CAG  CTG TTT GGT AGG GAG  CTG TTT GGT AGG CGG  CTG TTT GGT AGG CAC  CTG TTT GCT ACG CAG |
|  |  |

**Table S2** The FAM-modified aptamers-Apt0, Apt8, Apt11 and Apt1-3.

| Aptamer number | Sequence(5'-3') |
| --- | --- |
| Apt0  Apt8  Apt11  Apt1-3 | 5'-FAM-CTG TTT GGT AGG CAG-3'  5'-FAM-CTG TTT GCT AGG CAG-3'  5'-FAM-CTG TTT GGT ACG CAG-3'  5'-FAM-CTG TTT GCT ACG CAG-3' |

**Table S3** Linear range, detection limit and molecular docking results for each mutant aptamer

(Linear range of Apt1-3 is 6.7-40.0 nmol/mL)

| Aptamer number | Regression equation  Linear Range (6.7-55.6 nmol/mL) | Coefficient  (R^2^) | LOD  (nmol/mL) | Binding Energy  (kal/mol) |
| --- | --- | --- | --- | --- |
| Apt0  Apt1  Apt2  Apt3  Apt4  Apt5  Apt6  Apt7  Apt8  Apt9  Apt10  Apt11  Apt12  Apt13  Apt14  Apt15  Apt1-3 | y = 0.01401x+0.01345  y = 0.01356x+0.0635  y = 0.01073x+0.0248  y=0.01385x-0.02423  y = 0.01342x+0.0054  y = 0.01349x+0.0285  y = 0.01155x+0.0209  y = 0.01243x+0.0372  y = 0.01831x-0.02201  y = 0.0122x+0.02536  y = 0.01275x+0.0330  y = 0.01738x-0.00542  y = 0.01188x+0.0137  y = 0.01171x+0.0092  y = 0.01355x-0.0035  y = 0.01184x+0.0675  y = 0.02895x-0.14316 | 0.9966  0.9959  0.9789  0.9851  0.9530  0.9775  0.9942  0.9641  0.9949  0.9896  0.9715  0.9954  0.9949  0.9818  0.9912  0.9729  0.9931 | 0.67  0.68  0.70  0.67  0.67  0.69  0.71  0.70  0.50  0.69  0.70  0.60  0.72  0.74  0.68  0.75  0.12 | -3.33  -3.24  -2.20  -3.20  -3.30  -3.31  -2.50  -3.32  -4.02  -2.60  -3.29  -3.48  -3.30  -2.60  -3.20  -3.30  -4.32 |


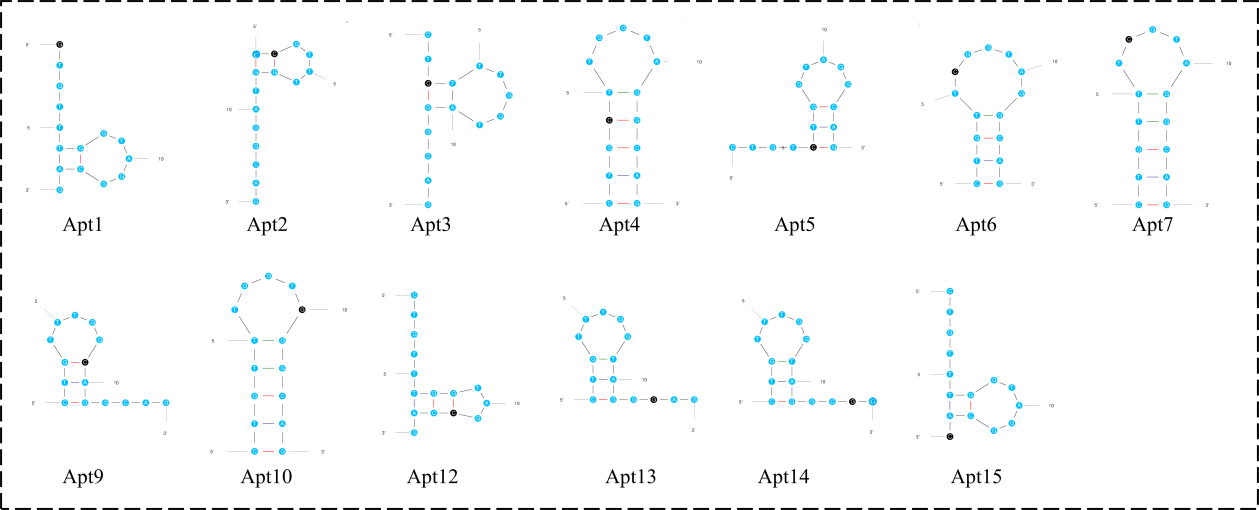


**Fig. S1** Secondary structure of aptamers obtained by base mutation.


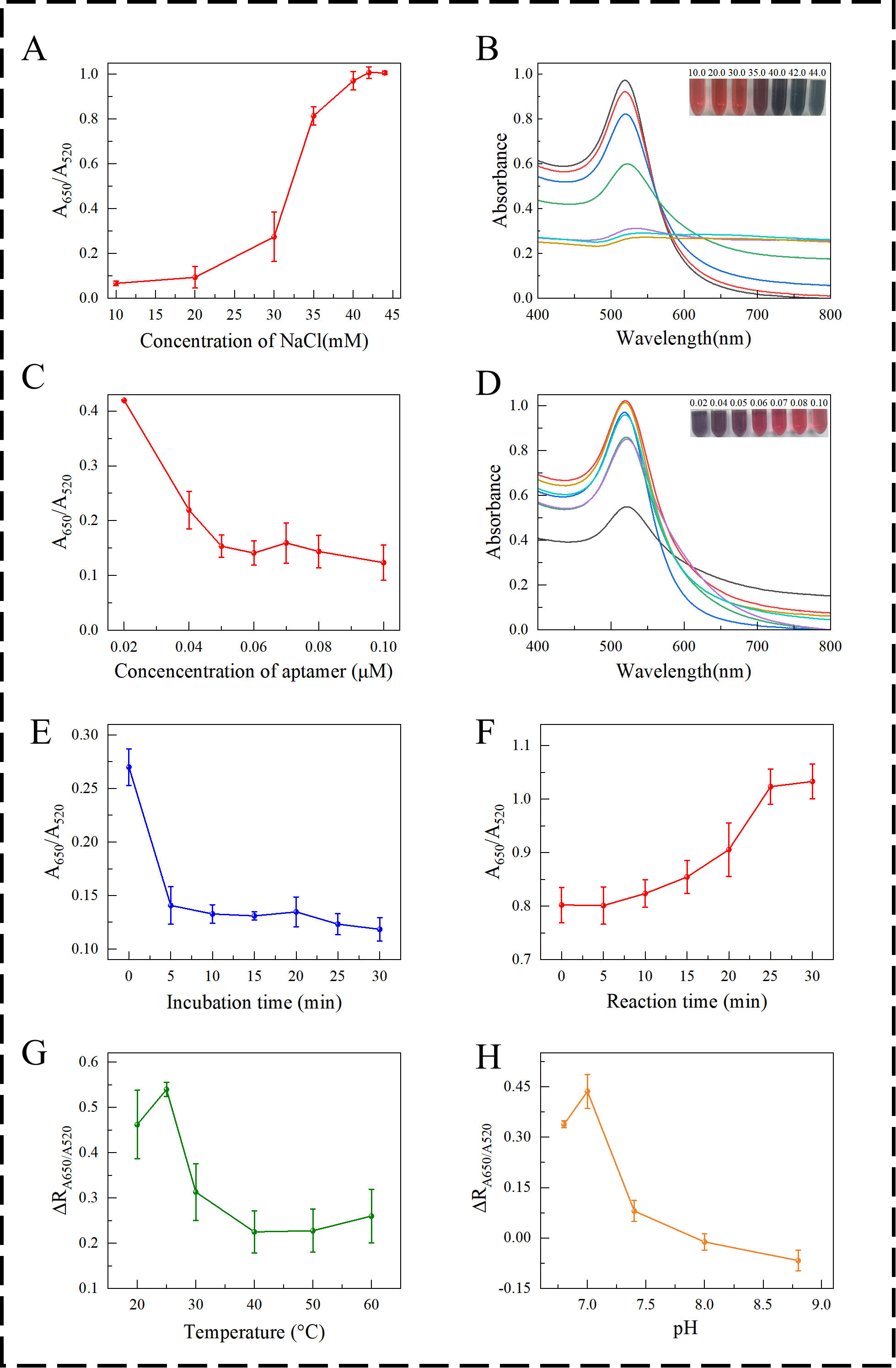
**Fig. S2** (A, B) The effect of NaCl concentration on the absorbance ratio of AuNPs in the sensor and UV absorption spectrum. (C, D) The effect of aptamer concentration on the absorbance ratio of AuNPs in the sensor and UV absorption spectrum. (E) The effect of aptamer and AuNPs incubation time on the UV absorbance ratio in the sensor. (F) The effect of reaction time of metronidazole and aptamer on the UV absorbance ratio in the sensor. (G) The effect of the temperature of the reaction system on the UV absorbance ratio in the sensor was studied. (H) The effect of the pH of the buffer solution on the UV absorbance ratio of the sensor was studied.


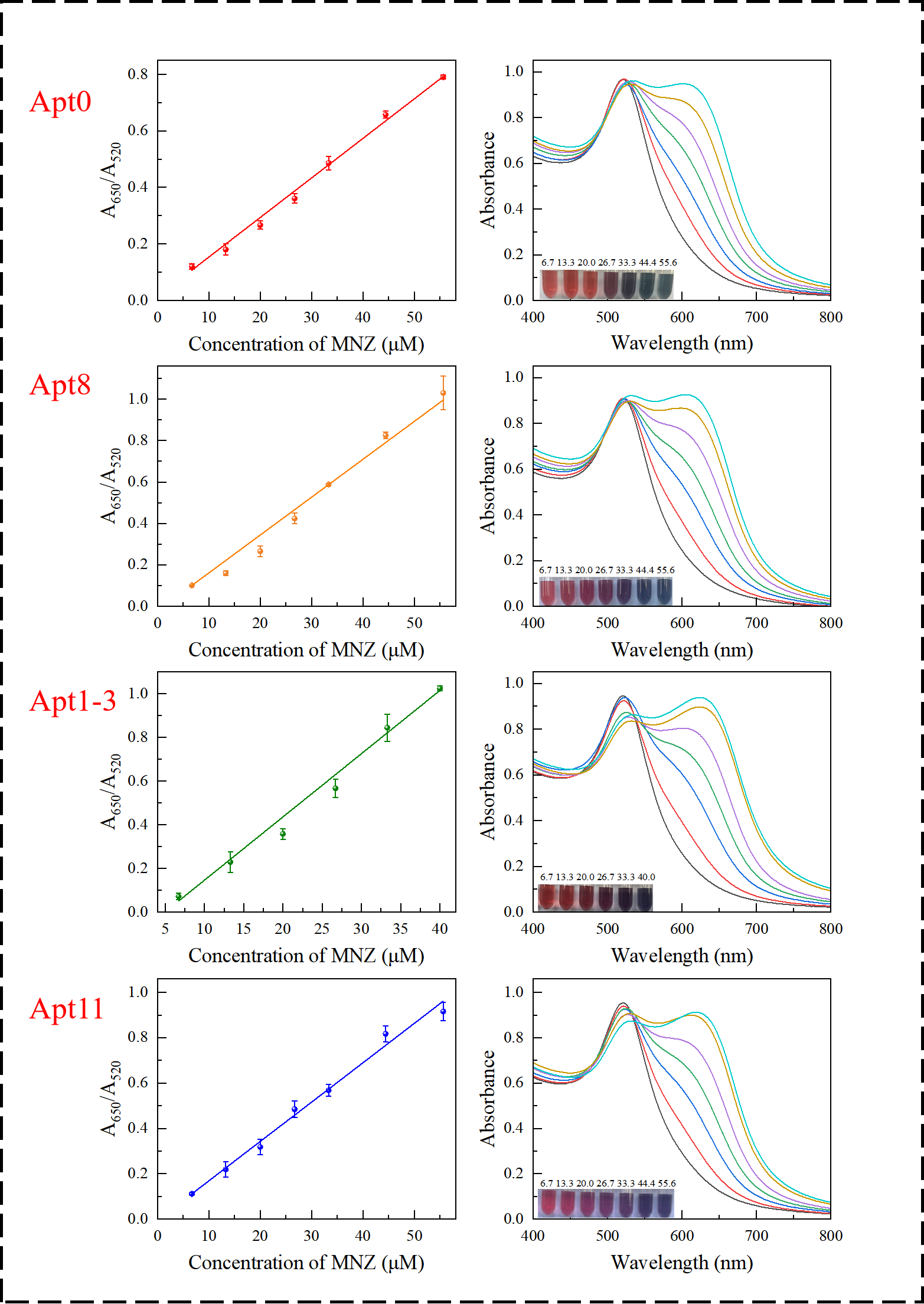


**Fig. S3** Apt0, Apt8, Apt11, and Apt1-3 Linear range and spectrogram.


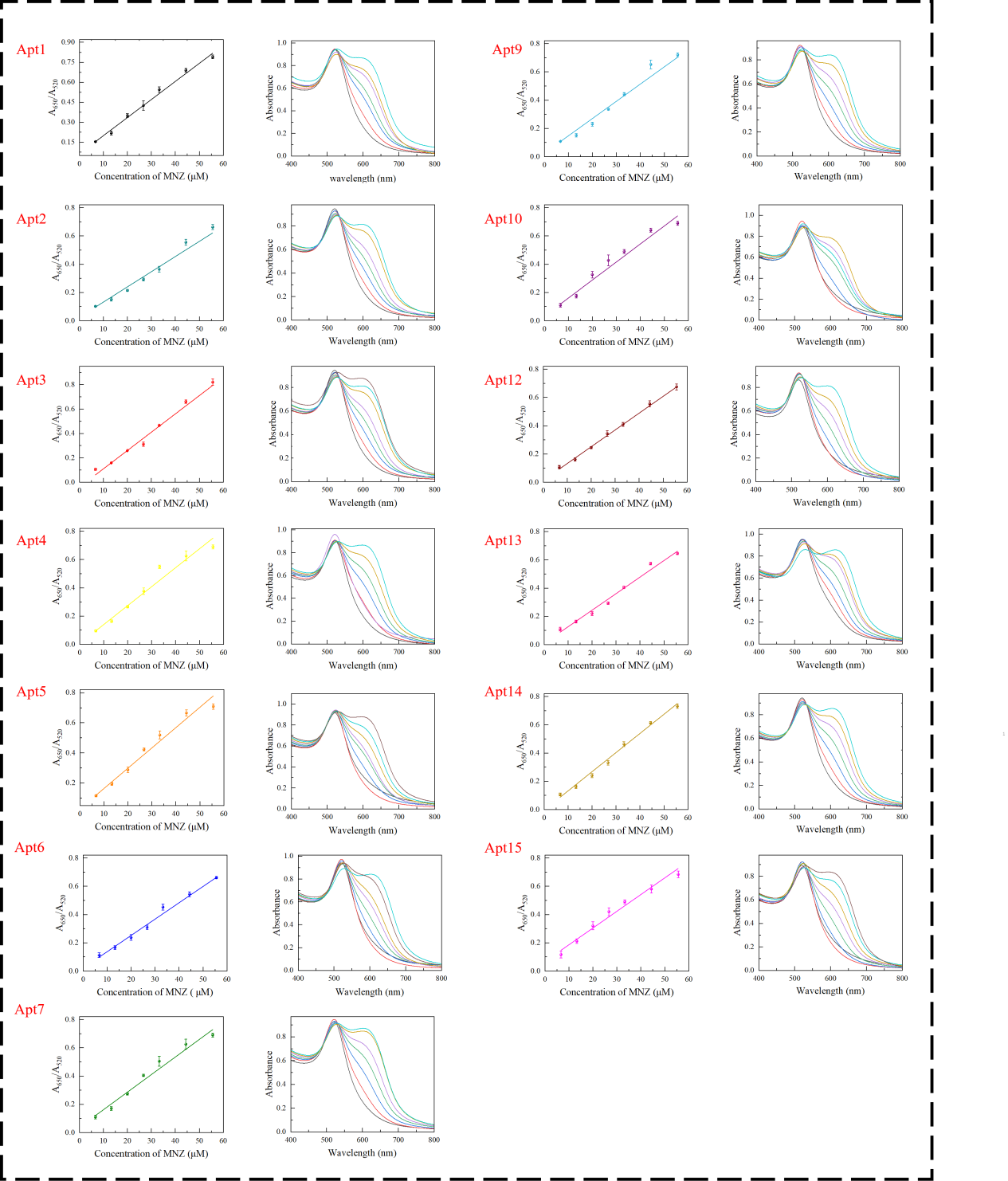


**Fig. S4** Linear range of aptamers obtained by base mutation and their spectra.

**Fig. S5** The design structure of the smartphone concealed box and its 3D display figure.

1. ﹡Corresponding authors. Tel: 18999250641 (J, Yao); 13999258239（M, Zhang）

   E-mail addresses: xydyaojun@163.com (J, Yao); zhang78089680@sina.com (M, Zhang)

   Sicheng Zhang, Yadi Qin and Jie Yuan contributed equally to this work. [↑](#footnote-ref-0)
2. [↑](#footnote-ref-1)
